# The effect of consolidation immunotherapy on patients with stage III non-small cell lung cancer who received induction chemoimmunotherapy: a multicenter retrospective study

**DOI:** 10.3389/fimmu.2026.1744239

**Published:** 2026-04-28

**Authors:** Jianian Lai, Song Guan, Yu Wang, Qingyu Wang, Meng Yan, Kai Ren, Jun Wang, Nan Bi, Lujun Zhao

**Affiliations:** 1 Department of Radiation Oncology, Tianjin’s Clinical Research Center for Cancer, Key Laboratory of Cancer Prevention and Therapy, Tianjin Medical University Cancer Institute & Hospital, National Clinical Research Center for Cancer, Tianjin, China; 2 Department of Radiation Oncology, Beijing Chest Hospital, Capital Medical University/Beijing Tuberculosis and Thoracic Tumor Research Institute, Beijing, China; 3 Department of Radiation Oncology, National Cancer Center/National Clinical Research Center for Cancer/Cancer Hospital, Chinese Academy of Medical Sciences and Peking Union Medical College, Beijing, China; 4 Department of Radiotherapy, The Fourth Hospital of Hebei Medical University, Hebei Clinical Research Center for Radiation Oncology, Shijiazhuang, China

**Keywords:** chemoradiotherapy, consolidation immunotherapy, induction chemoimmunotherapy, non-small cell lung cancer, prognosis

## Abstract

**Purpose:**

The purpose of this study was to evaluate the effect of consolidation immunotherapy on patients with stage III non-small cell lung cancer (NSCLC) who received induction chemoimmunotherapy before chemoradiotherapy (CRT).

**Materials and methods:**

Patients with stage III NSCLC who received induction chemoimmunotherapy before CRT with or without consolidation immunotherapy at 4 hospitals between February 2018 and December 2022 were retrospectively analyzed. The patients were divided into two groups on the basis of whether they received consolidation immunotherapy (Ind+Con group) or not (Ind group). Progression-free survival (PFS) and overall survival (OS) were assessed from the initiation of treatment and were estimated using the Kaplan–Meier method. One-to-one propensity score matching (PSM) was used to further minimize confounding effects.

**Results:**

A total of 196 eligible patients were enrolled, with 124 (63.3%) in the Ind group and 72 (36.7%) in the Ind+Con group. The median follow-up was 24.6 months, and the median PFS and OS for the whole cohort were 24.8 months and 46.0 months, respectively. The median PFS was 25.5 months in the Ind group vs. 24.0 months in the Ind+Con group, with 2-year PFS rates of 52.2% vs. 47.7% (P = 0.472). The median OS was 46.0 months in the Ind group vs. not reached (NR) in the Ind+Con group, with 2-year OS rates of 78.0% vs. 83.8% (P = 0.578). After 1:1 PSM, the median PFS was 30.2 months vs. 24.0 months, with 2-year PFS rates of 55.4% vs. 47.7% (P = 0.261). The median OS was 46.0 months vs. NR, with 2-year OS rates of 80.8% vs. 83.8% (P = 0.960).

**Conclusion:**

The effect of consolidation immunotherapy on patients with stage III NSCLC who receive induction chemoimmunotherapy before CRT needs to be further studied.

## 1 Introduction

The advent of immunotherapy has revolutionized the treatment paradigm for stage III non-small cell lung cancer (NSCLC). For patients with unresectable stage III NSCLC, the standard of care is concurrent chemoradiotherapy (cCRT) followed by consolidation immunotherapy (the PACIFIC regimen) (1, 2). However, the 2-year progression-free survival (PFS) rate in the immunotherapy group was only 45.0%, indicating that more than 50% of patients experienced disease progression even with consolidation immunotherapy (2). Given the promising efficacy of induction chemoimmunotherapy in patients with resectable stage III NSCLC, with a 2-year event-free survival (EFS) rate of 63.8% (3), induction immunotherapy before chemoradiotherapy (CRT) is recommended for patients with bulky disease or extensive lymph node metastasis to reduce the tumor burden and increase the likelihood of success with subsequent definitive CRT (4–6). Our preliminary studies suggested that in patients with stage III NSCLC, induction immunotherapy could significantly improve the outcomes of CRT (7) and achieve comparable outcomes to those of consolidation immunotherapy (8). The combination of induction plus consolidation immunotherapy has demonstrated favorable safety and efficacy and has been shown to have greater protective effects against distant metastasis than consolidation immunotherapy alone (9–11). At the same time, a recent prospective phase II trial (CA209-7AL) demonstrated that consolidative nivolumab following neoadjuvant nivolumab plus chemotherapy and cCRT significantly prolonged PFS compared with observation (12). However, the trial included only patients who received cCRT, and its applicability to those treated with sequential chemoradiotherapy (sCRT) in routine practice is uncertain. Moreover, the recent debate over the necessity of postoperative adjuvant immunotherapy after preoperative neoadjuvant chemoimmunotherapy has raised the question of whether patients who have already received chemoimmunotherapy before CRT would benefit from combined consolidation immunotherapy (13–15). Therefore, we conducted this multicenter retrospective study to evaluate whether consolidation immunotherapy provides additional benefit after induction chemoimmunotherapy in patients with unresectable stage III NSCLC using real-world data.

## 2 Materials and methods

### 2.1 Patient selection

Patients with unresectable stage III NSCLC who received induction chemoimmunotherapy before CRT with or without consolidation immunotherapy after CRT at Tianjin Medical University Cancer Institute & Hospital, National Cancer Center/National Clinical Research Center for Cancer/Cancer Hospital, Beijing Chest Hospital, and the Fourth Hospital of Hebei Medical University between February 2018 and December 2022 were included in this multicenter, retrospective study. The inclusion criteria were as follows: 1) age ≥18 years; 2) histopathologically confirmed stage III NSCLC; and 3) completion of induction chemoimmunotherapy before thoracic radiation, with or without subsequent consolidation immunotherapy. The exclusion criteria included the following: 1) the presence of mutant driver genes, such as epidermal growth factor receptor (EGFR) mutations or anaplastic lymphoma kinase (ALK) rearrangements; 2) a history of any cancer-specific treatment; 3) a history of induction therapy with immune checkpoint inhibitor (ICI) monotherapy; and 4) a history of immunotherapy concurrent with radiotherapy. Baseline characteristics and therapeutic information were extracted from medical records. Histological types and disease stages were determined according to the WHO criteria (16) and the International Association for the Study of Lung Cancer classification (8^th^ edition) (17), respectively.

According to the sequence of immunotherapy and radiotherapy, patients were classified into an induction chemoimmunotherapy (Ind) group and an induction chemoimmunotherapy plus consolidation immunotherapy (Ind+Con) group. This study conformed to the provisions of the Declaration of Helsinki (as revised in 2013) and was approved by the institutional medical ethics committee (bc2022212).

### 2.2 Treatment

The treatment regimen for each patient was determined by a multidisciplinary team comprising surgeons and radiation oncologists. Induction chemoimmunotherapy was administered primarily for patients with initially resectable stage II-III NSCLC or for patients who were going to receive definitive CRT to reduce target volumes that were too large or too extensive to meet the normal tissue constraints. All patients were treated with PD-1/PD-L1 inhibitors, such as camrelizumab, nivolumab, pembrolizumab, sintilimab, tislelizumab, or toripalimab. Patients who developed grade 3–4 adverse events were managed in accordance with national and international guidelines, typically requiring interruption or permanent discontinuation of immunotherapy and initiation of high-dose corticosteroids, with additional immunosuppressive agents when indicated. All patients received platinum-based doublet chemotherapy administered in 21-day cycles (see **Supplementary Table 1**). Chemotherapy regimens were selected primarily on the basis of tumor histological type and individual patient characteristics. Radiotherapy was delivered using intensity-modulated radiation therapy (IMRT), with a total dose of 45–66 Gy administered in 15–33 fractions (see **Supplementary Data 1**).

### 2.3 Study outcomes

The primary clinical outcomes were PFS and overall survival (OS). PFS was defined as the time from treatment initiation to the first documented disease progression, death from any cause without prior progression, or last follow-up. OS was defined as the time from treatment initiation to death from any cause or last follow-up. Patients underwent follow-up visits every 3 months for the first 2 years and every 6 months thereafter. These visits included clinical evaluation, computed tomography (CT) or positron emission tomography (PET), and additional investigations when clinically indicated. Treatment-related adverse events (TRAEs) were graded according to CTCAE version 5.0. Objective response rates (ORRs) and disease control rates (DCRs) were evaluated following induction chemoimmunotherapy. In accordance with RECIST v1.1, the ORR was defined as a complete or partial response (CR or PR), while the DCR was defined as CR, PR, or stable disease (SD).

### 2.4 Statistical analysis

Patient characteristics were compared between treatment groups using the chi-square test or Fisher’s exact test for categorical variables and the Wilcoxon rank-sum test for continuous variables. PFS and OS were estimated by the Kaplan–Meier method and compared with the log-rank test. To reduce potential confounding, one-to-one propensity score matching (PSM) with a caliper of 0.02 was performed based on the following baseline variables, including age (< 65 vs. ≥ 65 years), sex (male vs. female), smoking history (never vs. former/current), Eastern Cooperative Oncology Group (ECOG) performance status (0, 1, 2), WHO histology type (squamous, non-squamous, not otherwise specified), stage (IIIA, IIIB, IIIC), CRT modality (sCRT vs. cCRT), radiation dose (< 54 vs. ≥ 54 Gy), induction immunotherapy cycles (≤ 4 vs. > 4), and response to induction chemoimmunotherapy (CR/PR vs. SD/progressive disease (PD)). Variables with P < 0.1 in univariate analysis were entered into the multivariable Cox proportional hazards model (Cox model). To examine the consistency of the treatment effect across subgroups, subgroup analyses for PFS were conducted using a non-stratified Cox model with treatment included as a covariate. To address potential immortal time bias due to differences in induction cycle numbers between the two groups, a sensitivity analysis was conducted with radiotherapy initiation defined as time zero. A P value less than 0.05 was considered to indicate statistical significance. Statistical analyses were performed using SPSS version 25 (IBM, Armonk, NY, USA).

## 3 Results

### 3.1 Baseline characteristics

**Figure 1:**
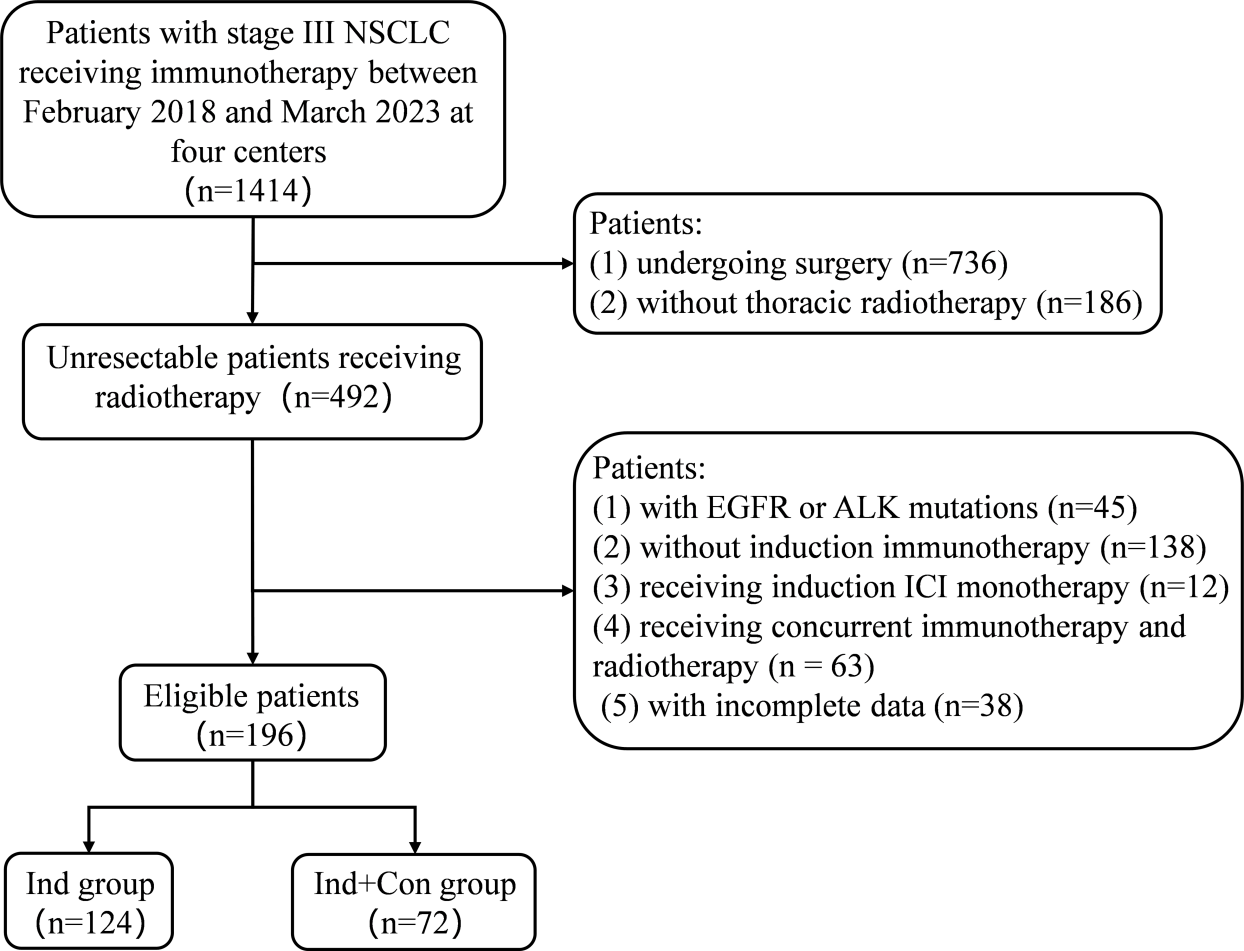
Patient inclusion flow chart.

A total of 196 consecutive eligible patients were enrolled in this study. Among them, 124 (63.3%) received induction chemoimmunotherapy alone, and 72 (36.7%) received additional consolidation immunotherapy (**Figure 1**). The median patient age was 63 years (range 27–79). The detailed clinical characteristics are shown in **Table 1**.

**Table 1:** Baseline characteristics of patients in the Ind and Ind+Con groups.

Characteristics	Before PSM			After PSM		
	Ind (n=124)	Ind+Con (n=72)		Ind (n=72)	Ind+Con (n=72)	
	No. (%)	No. (%)	P	No. (%)	No. (%)	P
Age						
< 65	62(50.0)	46(63.9)	0.059	36(50.0)	46(63.9)	0.092
≥ 65	62(50.0)	26(36.1)		36(50.0)	26(36.1)	
Sex						
Male	108(87.1)	69(95.8)	0.046	69(95.8)	69(95.8)	1.000
Female	16(12.9)	3(4.2)		3(4.2)	3(4.2)	
Smoking history						
Never	25(20.2)	7(9.7)	0.057	12(16.7)	7(9.7)	0.218
Former/Current	99(79.8)	65(90.3)		60(83.3)	65(90.3)	
ECOG						
0	9(7.3)	4(5.6)	0.674	4(5.6)	4(5.6)	0.632
1	106(85.5)	65(90.3)		62(86.1)	65(90.3)	
2	9(7.3)	3(4.2)		6(8.3)	3(4.2)	
WHO histology						
Squamous	93(75.0)	55(76.4)	0.958	57(79.2)	55(76.4)	0.894
Non-squamous	26(21.0)	14(19.4)		13(18.1)	14(19.4)	
NOS	5(4.0)	3(4.2)		2(2.8)	3(4.2)	
Stage						
IIIA	45(36.3)	18(25.0)	0.096	21(29.2)	18(25.0)	0.701
IIIB	54(43.5)	43(59.7)		43(59.7)	43(59.7)	
IIIC	25(20.2)	11(15.3)		8(11.1)	11(15.3)	
CRT modality						
sCRT	92(74.2)	49(68.1)	0.357	51(70.8)	49(68.1)	0.717
cCRT	32(25.8)	23(31.9)		21(29.2)	23(31.9)	
Radiation dose						
< 54 Gy	19(15.3)	12(16.7)	0.804	7(9.7)	12(16.7)	0.218
≥ 54 Gy	105(84.7)	60(83.3)		65(90.3)	60(83.3)	
Ind cycles						
≤ 4	83(66.9)	51(70.8)	0.572	50(69.4)	51(70.8)	0.856
> 4	41(33.1)	21(29.2)		22(30.6)	21(29.2)	
ICI target						
PD-1	124(100.0)	70(97.2)	0.259	72(100.0)	70(97.2)	0.476
PD-L1	0(0.0)	2(2.8)		0(0.0)	2(2.8)	
Response						
CR/PR	82(66.1)	53(73.6)	0.275	56(77.8)	53(73.6)	0.560
SD/PD	42(33.9)	19(26.4)		16(22.2)	19(26.4)	

Ind, induction chemoimmunotherapy; Con, consolidation immunotherapy; PSM, propensity score matching; ECOG, Eastern Cooperative Oncology Group; NOS, not otherwise specified; CRT, chemoradiotherapy; sCRT, sequential chemoradiotherapy; cCRT, concurrent chemoradiotherapy; Ind cycles, induction immunotherapy cycles; ICI, immune checkpoint inhibitor; Response, response to induction therapy; CR, complete response; PR, partial response; SD, stable disease; PD, progressive disease.

### 3.2 Treatment

The ICI agents used included camrelizumab (13.8%, n=27), nivolumab (3.6%, n=7), pembrolizumab (25.5%, n=50), sintilimab (33.2%, n=65), tislelizumab (20.9%, n=41), and toripalimab (3.1%, n=6). All patients in the Ind group received induction ICI plus chemotherapy, with a median of 4 cycles of induction immunotherapy (range 1–12; **Supplementary Figure 1**). Only four patients received more than eight cycles of induction immunotherapy. Patients in the Ind+Con group received induction ICI plus chemotherapy followed by consolidation immunotherapy, with a median of 4 cycles of induction immunotherapy (range 1–8; **Supplementary Figure 1**) and 7 cycles of consolidation immunotherapy (range 1–50).

### 3.3 Treatment efficacy

In the entire cohort, the median follow-up from the initiation of treatment was 24.6 months (range 5.7–54.1 months). The median PFS and OS were 24.8 and 46.0 months, respectively. During follow-up, 96 patients developed PD, including 59 (47.6%) in the Ind group and 37 (51.4%) in the Ind+Con group. Forty-three patients died at the time of analysis, including 29 (23.4%) and 14 (19.4%) patients in the two groups, respectively. The majority of patients (72.1%) died of lung cancer, with the remaining causes of death detailed in **Supplementary Table 2**.

**Figure 2:**
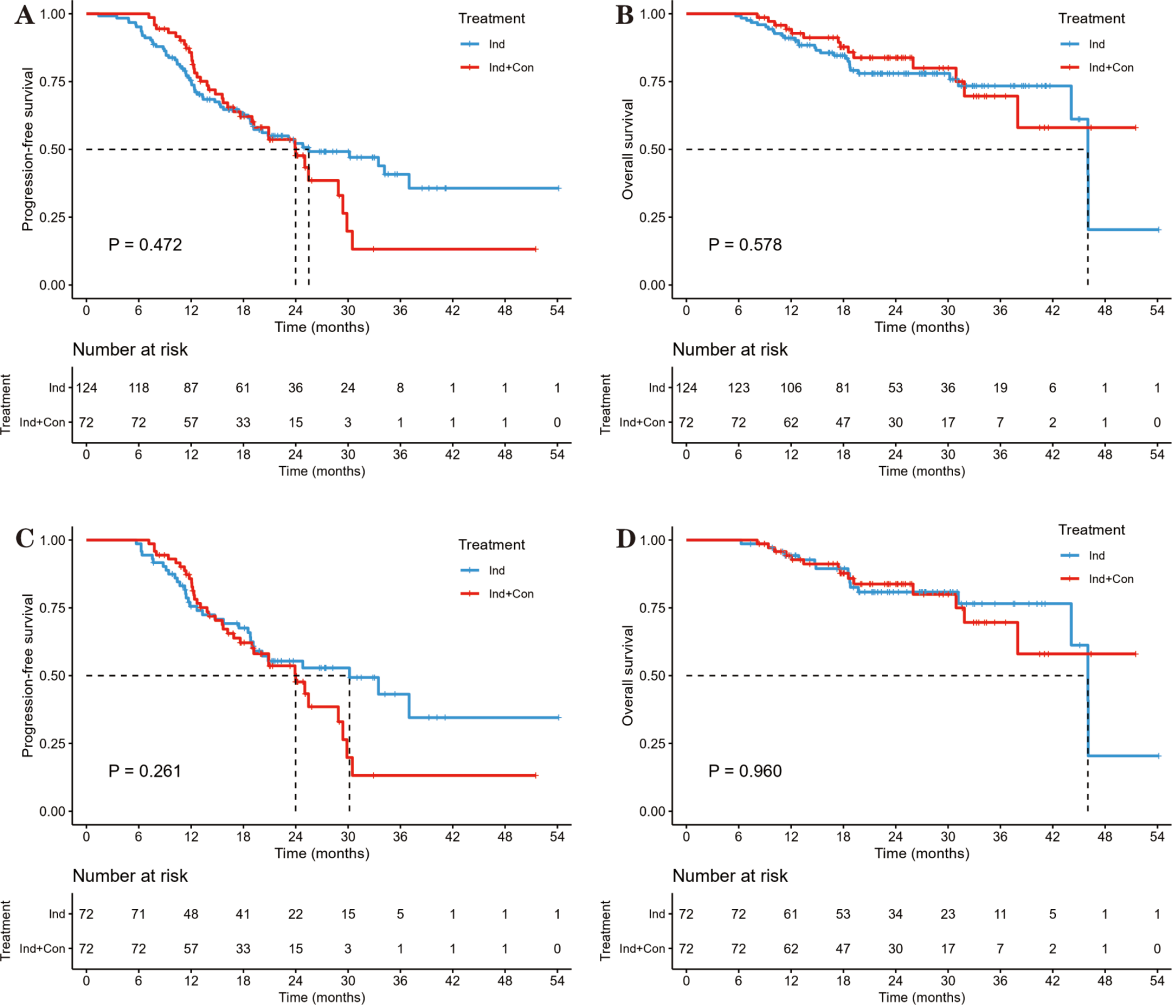
PFS and OS between the Ind and Ind+Con groups before and after PSM, measured from the initiation of induction therapy. **(A)** PFS before PSM. **(B)** OS before PSM. **(C)** PFS after PSM. **(D)** OS after PSM.

**Figure 3:**
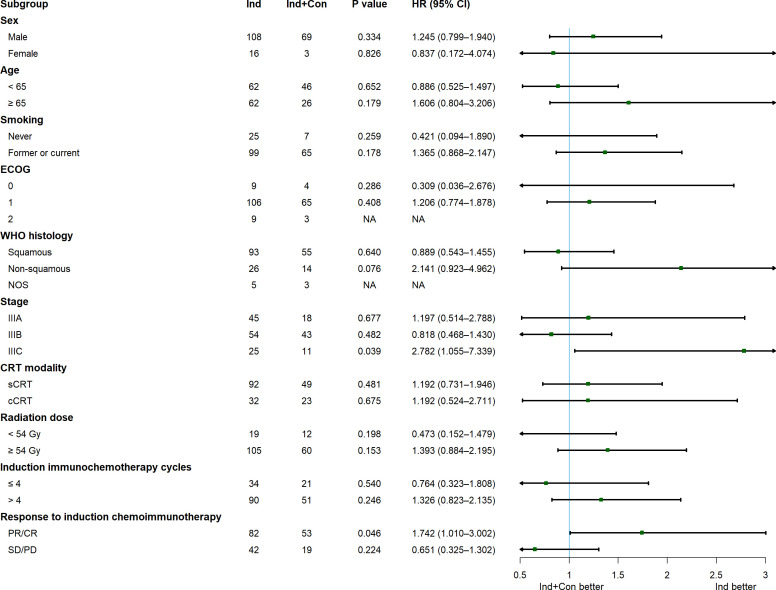
Subgroup analysis of the effect of consolidation immunotherapy on PFS in the overall cohort.

The median PFS was 25.5 months in the Ind group vs. 24.0 months in the Ind+Con group, with 1-year PFS rates of 75.5% vs. 85.7% and 2-year PFS rates of 52.2% vs. 47.7% (P = 0.472; **Figure 2A**). The median OS was 46.0 months in the Ind group vs. not reached (NR) in the Ind+Con group, with 1-year OS rates of 91.0% vs. 94.3% and 2-year OS rates of 78.0% vs. 83.8% (P = 0.578; **Figure 2B**). Univariate and multivariate analyses further revealed the limited value of additional consolidation immunotherapy in improving PFS (HR = 1.167, P = 0.467; **Supplementary Table 3**) and OS (HR = 0.763, P = 0.421; **Supplementary Table 4**). Subgroup analyses showed largely comparable PFS between the two groups, except in patients with stage IIIC (P = 0.039) and those who achieved CR or PR after induction chemoimmunotherapy (P = 0.046), with PFS favoring the Ind group in both subgroups (**Figure 3**).

After further 1:1 PSM, 72 matched pairs from both groups were included in the final analysis. The balance was assessed using standardized mean differences (SMDs), all of which were < 0.2, indicating good covariate balance (**Supplementary Figure 2**). The survival rates for the two treatment modalities were similar. The median PFS was 30.2 months vs. 24.0 months, with 1- and 2-year PFS rates of 75.6% vs. 85.7% and 55.4% vs. 47.7% (P = 0.261; **Figure 2C**). The median OS was 46.0 months vs. NR, with corresponding 1- and 2-year OS rates of 94.3% vs. 94.3% and 80.8% vs. 83.8% (P = 0.960; **Figure 2D**).

The incidence of pneumonitis was significantly lower in the Ind group than in the Ind+Con group (54.8% vs. 69.4%, P = 0.044; **Table 2**), while the incidence of esophagitis was similar between the two groups (25.8% vs. 36.1%, P = 0.128; **Table 2**).

**Table 2:** TRAEs between the Ind and Ind+Con groups.

TRAE	Ind		Ind+Con		
	No.	%	No.	%	P
Whole population					
Pneumonitis	68	54.8	50	69.4	0.044
G3/4 pneumonitis	10	8.1	12	16.7	0.066
Esophagitis	32	25.8	26	36.1	0.128
G3/4 esophagitis	1	0.8	0	0.0	1.000
Hematologic toxicity	67	54.0	33	45.8	0.268
G3/4 hematologic toxicity	19	15.3	10	13.9	0.785
Dermatitis	7	5.6	5	6.9	0.955
G3/4 dermatitis	2	1.6	1	1.4	1.000
Peripheral neuropathy	2	1.6	1	1.4	1.000
Abnormal liver function	1	0.8	0	0.0	1.000
Hypothyroidism	1	0.8	0	0.0	1.000
Capillary hemangiomas	1	0.8	1	1.4	1.000
Response: CR/PR					
Pneumonitis	49	59.8	38	71.7	0.157
G3/4 pneumonitis	7	8.5	10	18.9	0.077
Esophagitis	21	25.6	20	37.7	0.135
G3/4 esophagitis	1	1.2	0	0.0	1.000
Hematologic toxicity	41	50.0	25	47.2	0.748
G3/4 hematologic toxicity	12	14.6	8	15.1	0.941
Dermatitis	7	8.5	5	9.4	1.000
G3/4 dermatitis	2	2.4	1	1.9	1.000
Peripheral neuropathy	1	1.2	1	1.9	1.000
Abnormal liver function	0	0.0	0	0.0	NA
Hypothyroidism	1	1.2	0	0.0	1.000
Capillary hemangiomas	1	1.2	1	1.9	1.000
Response: SD/PD					
Pneumonitis	19	45.2	12	63.2	0.195
G3/4 pneumonitis	3	7.1	2	10.5	1.000
Esophagitis	11	26.2	6	31.6	0.664
G3/4 esophagitis	0	0.0	0	0.0	NA
Hematologic toxicity	26	61.9	8	42.1	0.149
G3/4 hematologic toxicity	7	16.7	2	10.5	0.813
Dermatitis	0	0.0	0	0.0	NA
G3/4 dermatitis	0	0.0	0	0.0	NA
Peripheral neuropathy	1	2.4	0	0.0	1.000
Abnormal liver function	1	2.4	0	0.0	1.000
Hypothyroidism	0	0.0	0	0.0	NA
Capillary hemangiomas	0	0.0	0	0.0	NA

TRAE, treatment-related adverse event; Ind, induction chemoimmunotherapy; Con, consolidation immunotherapy; G3/4, grade 3/4; Response, response to induction therapy; CR, complete response; PR, partial response; SD, stable disease; PD, progressive disease; NA, not available.

### 3.4 Exploratory analysis

**Figure 4:**
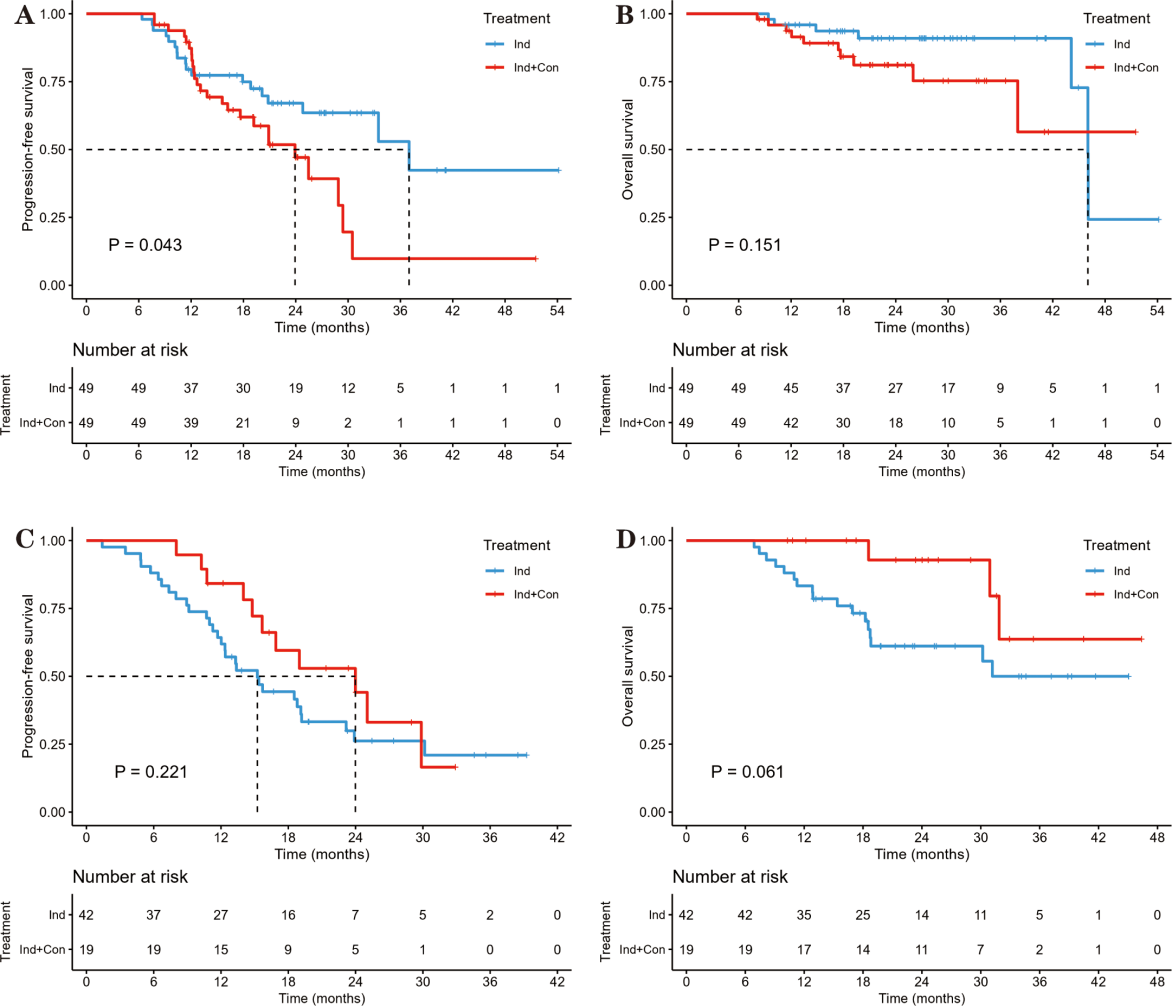
PFS and OS between the Ind and Ind+Con groups stratified by response to induction chemoimmunotherapy, measured from the initiation of induction therapy. **(A)** PFS in patients with CR or PR. **(B)** OS in patients with CR or PR. **(C)** PFS in patients with SD or PD. **(D)** OS in patients with SD or PD.

The ORR and DCR after induction chemoimmunotherapy were 68.9% and 97.4%, respectively. Only five (2.6%) patients progressed during the induction chemoimmunotherapy phase: four with local progression and one with systemic progression. When patients were stratified by their response to induction chemoimmunotherapy, the addition of consolidation immunotherapy appeared to have different effects in responders (CR/PR) than in non-responders (SD/PD). In the responders, the median PFS was 37.0 months in the Ind group vs. 23.9 months in the Ind+Con group, with 2-year PFS rates of 67.1% vs. 47.1% (P = 0.043; **Figure 4A**). The median OS was 46.0 months in the Ind group vs. NR in the Ind+Con group, with 2-year OS rates of 91.0% vs. 81.1% (P = 0.151; **Figure 4B**). In contrast, among patients with SD or PD, the median PFS was 15.2 months vs. 24.0 months, with 2-year PFS rates of 26.2% vs. 44.1% (P = 0.221; **Figure 4C**). The median OS remained unreached in both groups, with 2-year OS rates of 61.1% vs. 92.9% (P = 0.061; **Figure 4D**). The incidence of TRAEs was similar between the two groups, regardless of the response to induction chemoimmunotherapy (**Table 2**).

To minimize the impact of CRT modality selection, we evaluated the clinical value of consolidation immunotherapy after induction chemoimmunotherapy in patients receiving sCRT and cCRT. In patients receiving sCRT, the median PFS was 30.2 months in the Ind group vs. 25.0 months in the Ind+Con group (P = 0.192; **Supplementary Figure 3A**), and the median OS was 44.1 months vs. NR (P = 0.700; **Supplementary Figure 3B**). In patients receiving cCRT, the median PFS was NR in the Ind group vs. 24.0 months in the Ind+Con group (P = 0.672; **Supplementary Figure 3C**), and the median OS was NR vs. 38.0 months (P = 0.892; **Supplementary Figure 3D**).

### 3.5 Sensitivity analysis

In the sensitivity analysis, six patients were excluded (five with disease progression before radiotherapy and one with a missing radiotherapy start date), leaving 190 patients for analysis. The median PFS was 26.3 months in the Ind group vs. 20.0 months in the Ind+Con group (P = 0.310; **Supplementary Figure 4A**), and the median OS was 42.0 months vs. NR (P = 0.573; **Supplementary Figure 4B**). Among the responders, the median PFS was 32.3 months vs. 19.1 months (P = 0.258; **Supplementary Figure 5A**). The median OS was NR in both groups (P = 0.614; **Supplementary Figure 5B**). For patients with SD or PD, the median PFS was 13.8 months vs. 20.0 months (P = 0.382; **Supplementary Figure 5C**). The median OS was NR in both groups (P = 0.037; **Supplementary Figure 5D**). In patients receiving sCRT, the median PFS was 20.3 months in the Ind group vs. 19.9 months in the Ind+Con group (P = 0.357; **Supplementary Figure 6A**), and the median OS was 42.1 months vs. NR (P = 0.546; **Supplementary Figure 6B**). In patients receiving cCRT, the median PFS was NR in the Ind group vs. 21.0 months in the Ind+Con group (P = 0.498; **Supplementary Figure 6C**), and the median OS was NR vs. 35.1 months (P = 0.992; **Supplementary Figure 6D**).

## 4 Discussion

Although neoadjuvant chemoimmunotherapy has demonstrated remarkable efficacy in patients with resectable NSCLC, approximately 20% of patients are unable to undergo surgery after neoadjuvant therapy (3, 18). Moreover, in real-world clinical practice, a considerable proportion of patients with large tumor burdens or those being treated with induction chemoimmunotherapy prior to surgical resection cannot undergo surgery and instead receive definitive CRT. The effect of consolidation immunotherapy on these patients remains unclear. To the best of our knowledge, this is the first multicenter, real-world study to evaluate the effect of consolidation immunotherapy after CRT following induction chemoimmunotherapy in stage III NSCLC patients and to directly compare outcomes between the two groups. To comprehensively capture the efficacy and safety of the entire treatment trajectory, we defined the initiation of induction therapy as time zero. Meanwhile, to reduce potential immortal time bias introduced by differences in induction cycles, we included the number of induction cycles in the Cox model and further performed a sensitivity analysis by redefining time zero as the initiation of radiotherapy. Our study revealed that the addition of consolidation immunotherapy failed to improve prognosis further and significantly increased the incidence of TRAEs, particularly treatment-related pneumonitis. The sensitivity analysis yielded results consistent with the primary analysis, lending support to the robustness of our conclusions. The survival benefits associated with consolidation immunotherapy may have been offset by its treatment-related side effects, and this hypothesis warrants further validation in future studies.

A previous study (19) suggested that consolidation immunotherapy was associated with a trend toward improved survival (median PFS, 21.9 vs. 23.8 months; P = 0.370; median OS, NR in both group; 2-year OS rates, 64.2% vs. 85.8%; P = 0.170). Another phase II randomized multicenter trial (12) also demonstrated survival benefits with the addition of consolidation immunotherapy. However, this study included a greater proportion of patients with favorable performance status (ECOG score of 0 in more than 60% of both groups), indicating that these patients were likely to have better treatment tolerance. Similarly, induction chemoimmunotherapy was administered for only two cycles. It also adopted a shorter-course hypofractionated radiotherapy regimen to allow patients to proceed with subsequent cCRT. These aspects differ substantially from those in our study and may have contributed to differences in both efficacy and safety outcomes. Nevertheless, as illustrated in **Figure 2D** of our study, consolidation immunotherapy may still offer potential benefits. Further research is warranted to identify the specific patient subgroups most likely to benefit from such treatment.

Notably, the use of cCRT in real-world practice is considerably lower than that in landmark trials such as the PACIFIC trial. Wu et al. reported that among 147 patients who received induction immunochemotherapy followed by definitive CRT, only 36.7% received cCRT (20). Similarly, another real-world study revealed that in patients who received induction chemoimmunotherapy followed by CRT, 61.5% received sCRT, and 38.5% received cCRT (7). These figures align closely with our cohort, in which the cCRT rate was approximately 30%. This gap underscores the disparity between trial populations and real-world practice, where patient heterogeneity, comorbidities, tumor burden, and physician preferences often preclude the use of standard cCRT in a substantial proportion of patients. In addition, we assessed the effect of consolidation immunotherapy separately in the cCRT and sCRT subgroups, and the findings were consistent with the overall cohort analysis.

When further stratified by response to induction chemoimmunotherapy, we found that the addition of consolidation immunotherapy may be necessary for non-responders. This finding is consistent with the results of an indirect meta-analysis (21), where the addition of adjuvant immunotherapy yielded a numerical improvement in EFS (HR 0.82, 95% CI 0.56–1.21; P = 0.32) for patients without a pathologic complete response (pCR), despite the absence of a significant OS benefit (HR 1.18, 95% CI 0.73–1.90; P = 0.51). Furthermore, the 2-year EFS rate of 93% and the 4-year OS rate of 95% among patients who achieved pCR in the CheckMate-816 study (22) suggest that induction chemoimmunotherapy alone may be sufficient for patients who respond well to immunotherapy. One reason could be that responders to induction chemoimmunotherapy may have already achieved most of the benefit after roughly 4 cycles, leaving little room for further improvement with consolidation. Moreover, subsequent radiotherapy may modify the tumor immune microenvironment (23), thereby enabling patients who respond poorly to induction chemoimmunotherapy to benefit from consolidation immunotherapy. Preclinical and translational studies have demonstrated that radiotherapy can induce immunogenic cell death, enhance antigen presentation, activate the cGAS–STING pathway, increase T-cell infiltration, and potentially generate systemic antitumor immune responses (the so-called abscopal effect) (24–26). These immunomodulatory effects may partially restore sensitivity to immune checkpoint blockade in patients with suboptimal responses to induction chemoimmunotherapy, providing a biological rationale for consolidation strategies in selected populations. It is important to note, however, that insensitivity to induction immunotherapy may not indicate true treatment resistance. This ambiguity mirrors the ongoing debate in the surgical setting regarding the necessity of postoperative immunotherapy after neoadjuvant chemoimmunotherapy, where it remains unclear which component drives the observed survival benefit (21, 27).

This study has several limitations. First, the inherent drawbacks of the retrospective design may limit the generalizability of the results. Furthermore, data concerning PD-L1 expression are incomplete because it is not routinely tested in stage III NSCLC at our four centers. In addition, our study did not further distinguish between different ICIs. Although a previous study demonstrated no statistically significant differences in safety or efficacy among various ICIs (28–31), subtle differences in pharmacodynamics and immune activation patterns cannot be entirely excluded; the same ICI agent should be used in future studies, and patients should be stratified by PD-L1 expression to minimize confounding effects. Second, the median follow-up of 24.6 months may be insufficient for a mature OS analysis, especially considering the potential long-tail effect of immunotherapy. As the median OS was NR in the Ind+Con group, longer follow-up is needed to determine whether the observed trends result in differences in OS. Thus, the current OS findings should be viewed as hypothesis-generating and not definitive. Third, detailed radiotherapy parameters—such as fractionation, target volume, and the interval between radiotherapy and immunotherapy—were not included in the analysis. These factors could impact efficacy and toxicity, and their absence may introduce confounding effects. Fourth, this analysis focused on survival outcomes and clinician-assessed toxicity, without including health-related quality of life (HRQoL) or patient-reported outcomes (PROs), which are crucial for long-term treatment strategies. Future prospective studies should incorporate HRQoL assessments to better define the risk-benefit balance of consolidation immunotherapy. Finally, the results may also be influenced by the relatively modest sample size. Despite these limitations, this is the first multicenter study to evaluate the role of consolidation immunotherapy after CRT following induction chemoimmunotherapy in stage III NSCLC using real-world data and directly compare them with each other.

## 5 Conclusions

The addition of consolidation immunotherapy appears to have an uncertain survival benefit for patients with unselected stage III NSCLC who have received induction chemoimmunotherapy before CRT. However, it may be beneficial for those who respond poorly to induction chemoimmunotherapy. The sensitivity analysis adjusting for immortal time bias yielded results consistent with the primary findings. These preliminary findings may provide some insights for clinical practice but should be further validated in head-to-head randomized clinical trials.

## Data Availability

The original contributions presented in the study are included in the article/**Supplementary Material**. Further inquiries can be directed to the corresponding authors.
