# Supplementary material for: The effect of consolidation immunotherapy on patients with stage III non-small cell lung cancer who received induction chemoimmunotherapy: a multicenter retrospective study
Source: Front Immunol. 2026 Apr 28;17:1744239. doi: 10.3389/fimmu.2026.1744239 (PMC13161075; doi:10.3389/fimmu.2026.1744239)
Supplement: Supplementary file 1 [file DataSheet1.docx]

Supplementary Material

# Supplementary Data

## Supplementary Data 1

CT simulation was performed, with the scan range covering the neck, chest, and upper abdomen. The gross tumor volume (GTV) included the radiographically visible primary tumor and lymph nodes positive before induction chemotherapy. For patients treated with a simultaneous integrated boost, the planning GTV (PGTV) was generated by adding a uniform 5-mm margin to the GTV in all directions; the clinical target volume (CTV) was created by a uniform 5-mm expansion of the GTV and included the nodal drainage regions of pre-induction positive nodes; and the planning target volume (PTV) was created by adding a uniform 5-mm margin to the CTV. Organs at risk were contoured according to an established atlas. Both lungs were contoured on lung windows and evaluated as a single structure, including small extrahilar vessels, with the pretreatment GTV, hila, and trachea/main bronchi excluded. The heart was contoured along the pericardium, from the inferior border of the main pulmonary artery to the cardiac apex. Prescription dose was 45–60 Gy in 15–30 fractions to the PTV, or (with simultaneous boost) 45–59.4 Gy in 25–33 fractions to the PTV and 50–66 Gy in 25–33 fractions to the PGTV.

# Supplementary Tables and Figures

## Supplementary Tables

Table S1 Chemotherapy regimen

| Chemotherapy regimen | Dosage |
| --- | --- |
| NP | Vinorelbine 25 mg/m² d1, d8; Cisplatin 75 mg/m² d1 |
| PP/ LP | Paclitaxel/Liposome-encapsulated paclitaxel 135~175 mg/m² d1; Cisplatin 75 mg/m² d1, or Carboplatin AUC=5~6 d1 |
| nab-PP | Nab-paclitaxel 100 mg/m² d1, d8, d15; Cisplatin 75mg/m² d1, or Carboplatin AUC=5~6 d1 |
| GP | Gemcitabine 1000~1250 mg/m² d1, d8; Cisplatin 75mg/m² d1, or Carboplatin AUC=5~6 d1 |
| DP | Docetaxel 60~75 mg/m² d1; Cisplatin 75 mg/m² d1, or Carboplatin AUC=5~6 d1 |
| AP | Pemetrexed 500 mg/m² d1; Cisplatin 75 mg/m² d1, or Carboplatin AUC=5~6 d1 |

Abbreviations: d1, Day 1; d8, Day 8; AUC, area under the plasma concentration–time curve; d15, Day 15.

Table S2 Causes of death

| Cause of death | Ind (n=29) | Ind+Con (n=14) |
| --- | --- | --- |
| Lung cancer | 21 (72.4) | 10 (71.4) |
| COVID-19 | 2 (6.9) | 0 (0.0) |
| Chronic disease | 2 (6.9) | 1 (7.1) |
| Pulmonary infection | 1 (3.4) | 0 (0.0) |
| Esophageal tracheal fistula | 1 (3.4) | 0 (0.0) |
| Cerebral infarction | 1 (3.4) | 0 (0.0) |
| Hemoptysis | 1 (3.4) | 1 (7.1) |
| Immune-related pneumonitis | 0 (0.0) | 1 (7.1) |
| Accident | 0 (0.0) | 1 (7.1) |

Abbreviations: Ind, induction chemoimmunotherapy; Con, consolidation immunotherapy; COVID-19, coronavirus disease 2019.

Table S3 Univariate and multivariate Cox regression analyses for PFS

| Factor | Univariate | |  | Multivariate | |  |
| --- | --- | --- | --- | --- | --- | --- |
|  | HR | 95% CI | P | HR | 95% CI | P |
| Age |  |  |  |  |  |  |
| < 65 | 1.000 (reference) | | |  | | |
| ≥ 65 | 0.724 | 0.479, 1.095 | 0.126 |  |  |  |
| Sex |  |  |  |  |  |  |
| Male | 1.000 (reference) | | |  | | |
| Female | 1.169 | 0.623, 2.195 | 0.627 |  |  |  |
| Smoking history |  |  |  |  |  |  |
| Never | 1.000 (reference) | | |  | | |
| Former/current | 1.003 | 0.577, 1.744 | 0.990 |  |  |  |
| ECOG |  |  |  |  |  |  |
| 0 | 1.000 (reference) | | |  | | |
| 1 | 0.861 | 0.398, 1.864 | 0.704 |  |  |  |
| 2 | 0.698 | 0.234, 2.080 | 0.519 |  |  |  |
| WHO histology |  |  |  |  |  |  |
| Squamous | 1.000 (reference) | | |  | | |
| Non-squamous | 0.948 | 0.590, 1.521 | 0.823 |  |  |  |
| NOS | 0.359 | 0.088, 1.466 | 0.153 |  |  |  |
| Stage |  |  |  |  |  |  |
| IIIA | 1.000 (reference) | | |  | | |
| IIIB | 1.283 | 0.805, 2.044 | 0.294 |  |  |  |
| IIIC | 1.120 | 0.610, 2.056 | 0.715 |  |  |  |
| CRT modality |  |  |  |  |  |  |
| sCRT | 1.000 (reference) | | |  | | |
| cCRT | 0.832 | 0.521, 1.330 | 0.443 |  |  |  |
| Radiation dose |  |  |  |  |  |  |
| <54Gy | 1.000 (reference) | | |  | | |
| ≥54Gy | 0.714 | 0.427, 1.193 | 0.199 |  |  |  |
| Ind cycles |  |  |  |  |  |  |
| ≤ 4 | 1.000 (reference) | | |  | | |
| > 4 | 1.209 | 0.801, 1.825 | 0.366 |  |  |  |
| Response |  |  |  |  |  |  |
| CR/PR | 1.000 (reference) | | | 1.000 (reference) | | |
| SD/PD | 1.965 | 1.311, 2.946 | 0.001 | 1.966 | 1.311, 2.947 | 0.001 |
| Treatment |  |  |  |  |  |  |
| Ind | 1.000 (reference) | | | 1.000 (reference) | | |
| Ind+Con | 1.165 | 0.768, 1.768 | 0.473 | 1.167 | 0.770, 1.770 | 0.467 |

Abbreviations: PFS, progression-free survival; HR, hazard ratio; ECOG, Eastern Cooperative Oncology Group; NOS, not otherwise specified; CRT, chemoradiotherapy; sCRT, sequential chemoradiotherapy; cCRT, concurrent chemoradiotherapy; Ind cycles, induction immunotherapy cycles; Response, response to induction therapy; CR, complete response; PR, partial response; SD, stable disease; PD, progressive disease; Ind, induction chemoimmunotherapy; Con, consolidation immunotherapy.

Table S4 Univariate and multivariate Cox regression analyses for OS

| Factor | Univariate | |  | Multivariate | |  |
| --- | --- | --- | --- | --- | --- | --- |
|  | HR | 95% CI | P | HR | 95% CI | P |
| Age |  |  |  |  |  |  |
| < 65 | 1.000 (reference) | | | 1.000 (reference) | | |
| ≥ 65 | 0.577 | 0.305, 1.093 | 0.092 | 0.584 | 0.303, 1.127 | 0.109 |
| Sex |  |  |  |  |  |  |
| Male | 1.000 (reference) | | |  | | |
| Female | 1.601 | 0.707, 3.628 | 0.259 |  |  |  |
| Smoking history |  |  |  |  |  |  |
| Never | 1.000 (reference) | | |  | | |
| Former/current | 1.335 | 0.556, 3.208 | 0.518 |  |  |  |
| ECOG |  |  |  |  |  |  |
| 0 | 1.000 (reference) | | |  | | |
| 1 | 1.762 | 0.422, 7.356 | 0.437 |  |  |  |
| 2 | 1.180 | 0.164, 8.489 | 0.869 |  |  |  |
| WHO histology |  |  |  |  |  |  |
| Squamous | 1.000 (reference) | | |  | | |
| Non-squamous | 1.015 | 0.508, 2.029 | 0.967 |  |  |  |
| NOS | 0.000 | 0.000 | 0.969 |  |  |  |
| Stage |  |  |  |  |  |  |
| IIIA | 1.000 (reference) | | |  | | |
| IIIB | 1.351 | 0.677, 2.695 | 0.393 |  |  |  |
| IIIC | 0.987 | 0.369, 2.640 | 0.980 |  |  |  |
| CRT modality |  |  |  |  |  |  |
| sCRT | 1.000 (reference) | | |  | | |
| cCRT | 1.150 | 0.589, 2.243 | 0.682 |  |  |  |
| Dose |  |  |  |  |  |  |
| < 54Gy | 1.000 (reference) | | |  | | |
| ≥ 54Gy | 0.319 | 0.164, 0.621 | <0.001 | 0.368 | 0.186, 0.731 | 0.004 |
| Ind cycles |  |  |  |  |  |  |
| ≤ 4 | 1.000 (reference) | | |  | | |
| > 4 | 1.174 | 0.631, 2.185 | 0.612 |  |  |  |
| Response |  |  |  |  |  |  |
| CR/PR | 1.000 (reference) | | | 1.000 (reference) | | |
| SD/PD | 2.013 | 1.104, 3.669 | 0.022 | 1.608 | 0.863, 2.997 | 0.135 |
| Treatment |  |  |  |  |  |  |
| Ind | 1.000 (reference) | | | 1.000 (reference) | | |
| Ind+Con | 0.834 | 0.440, 1.581 | 0.578 | 0.763 | 0.394, 1.476 | 0.421 |

Abbreviations: OS, overall survival; HR, hazard ratio; ECOG, Eastern Cooperative Oncology Group; NOS, not otherwise specified; CRT, chemoradiotherapy; sCRT, sequential chemoradiotherapy; cCRT, concurrent chemoradiotherapy; Ind cycles, induction immunotherapy cycles; Response, response to induction therapy; CR, complete response; PR, partial response; SD, stable disease; PD, progressive disease; Ind, induction chemoimmunotherapy; Con, consolidation immunotherapy.

## Supplementary Figures


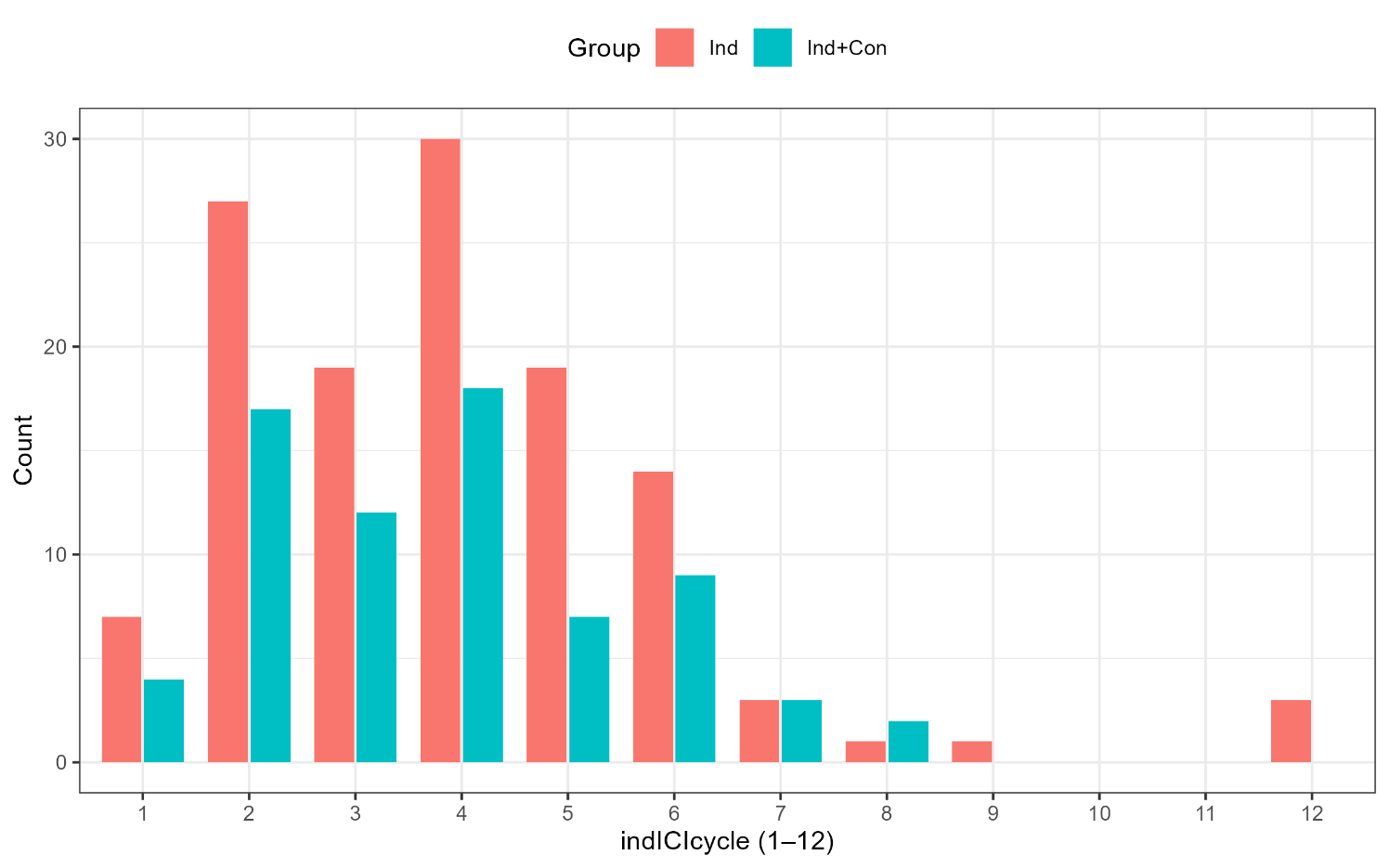


**Figure S1.** Distribution of induction immunotherapy cycles in the Ind and Ind+Con groups shown as bar plots.


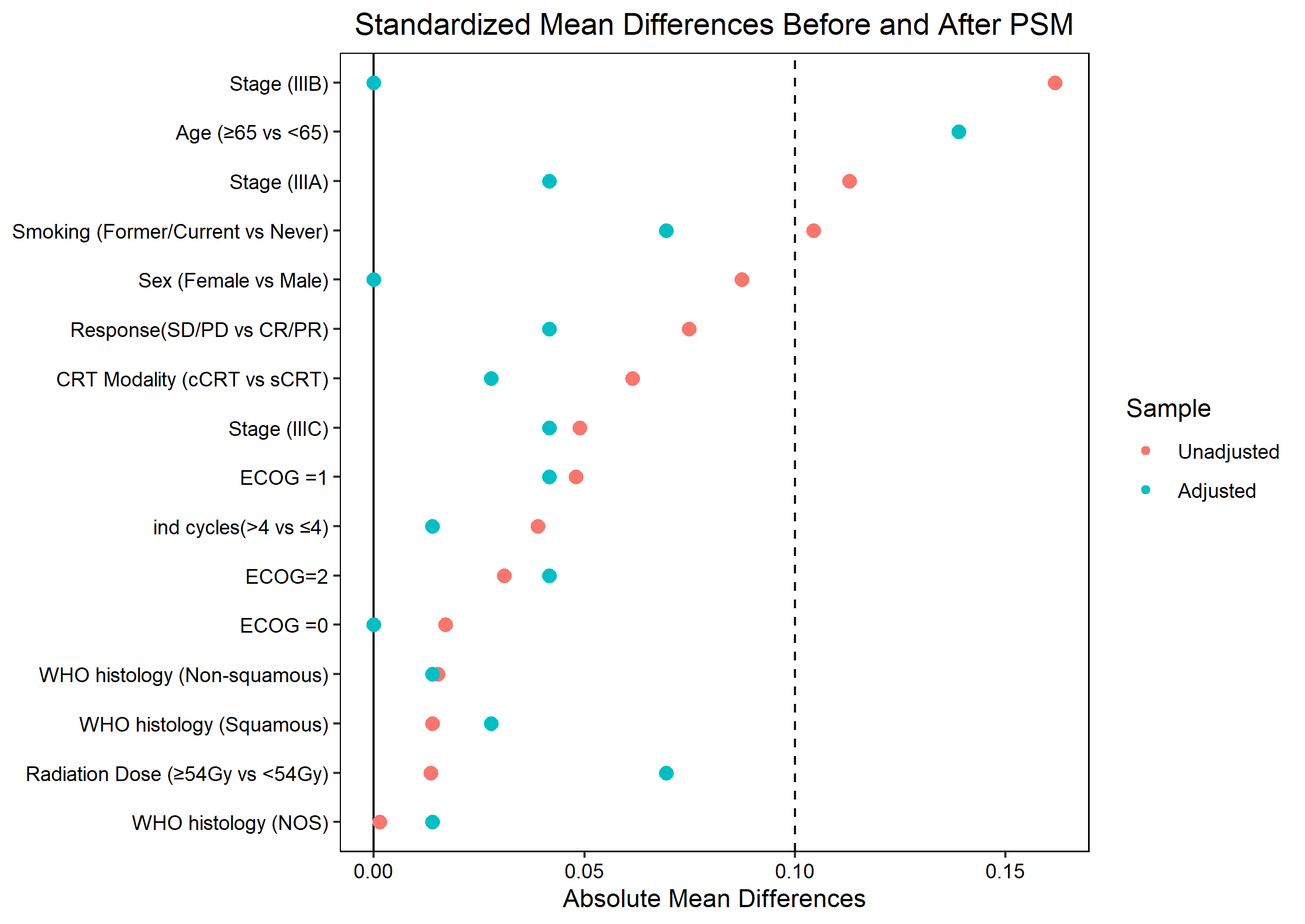


**Figure S2.** Love plot showing SMD of baseline characteristics before and after 1:1 PSM between the Ind and Ind+Con groups.


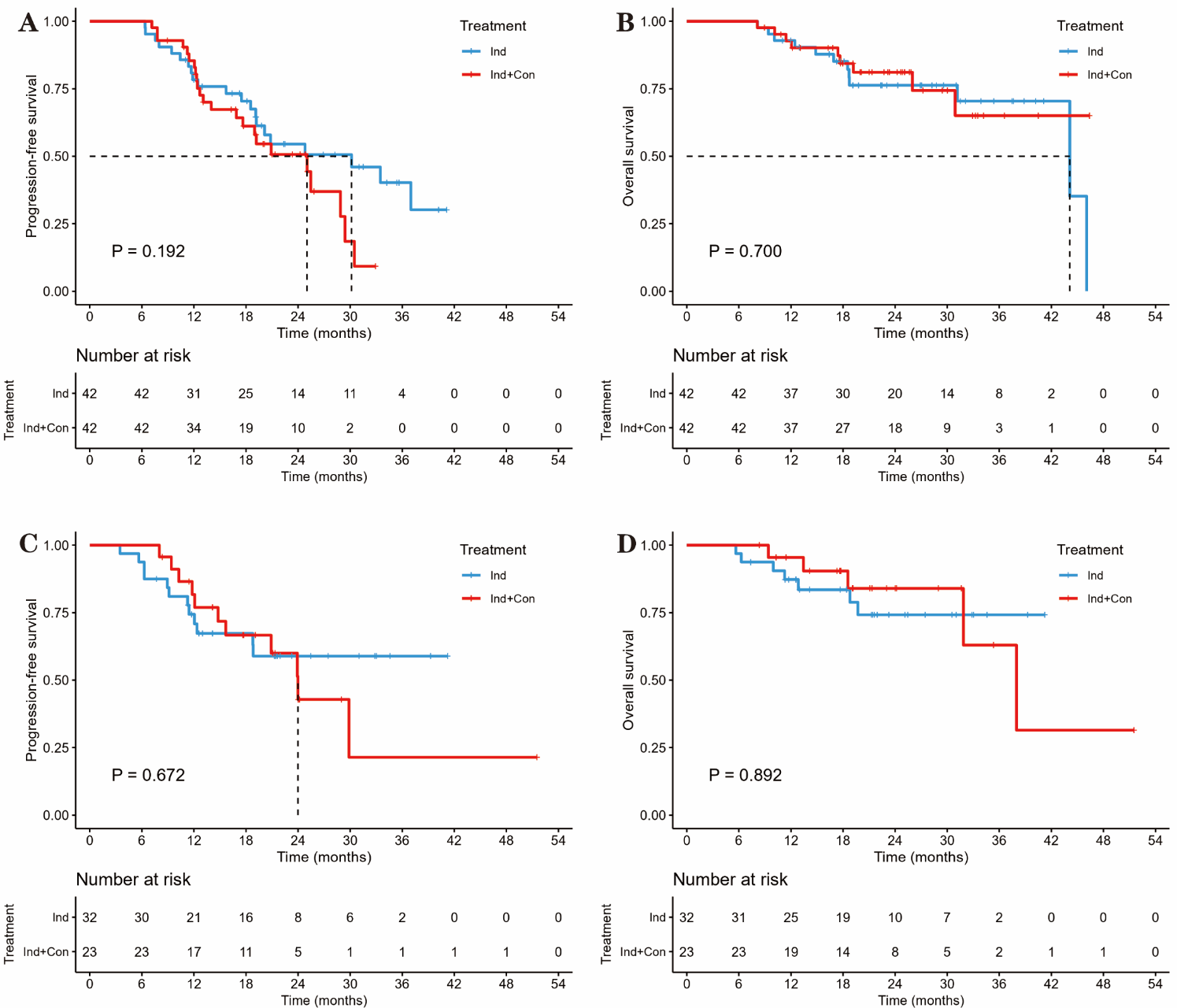


**Figure S3.** PFS and OS between the Ind and Ind+Con groups in the sequential or concurrent chemoradiotherapy subgroup, measured from the initiation of induction therapy. **(A)** PFS in the sequential chemoradiotherapy subgroup. **(B)** OS in the sequential chemoradiotherapy subgroup. **(C)** PFS in the concurrent chemoradiotherapy subgroup. **(D) O**S in the concurrent chemoradiotherapy subgroup.


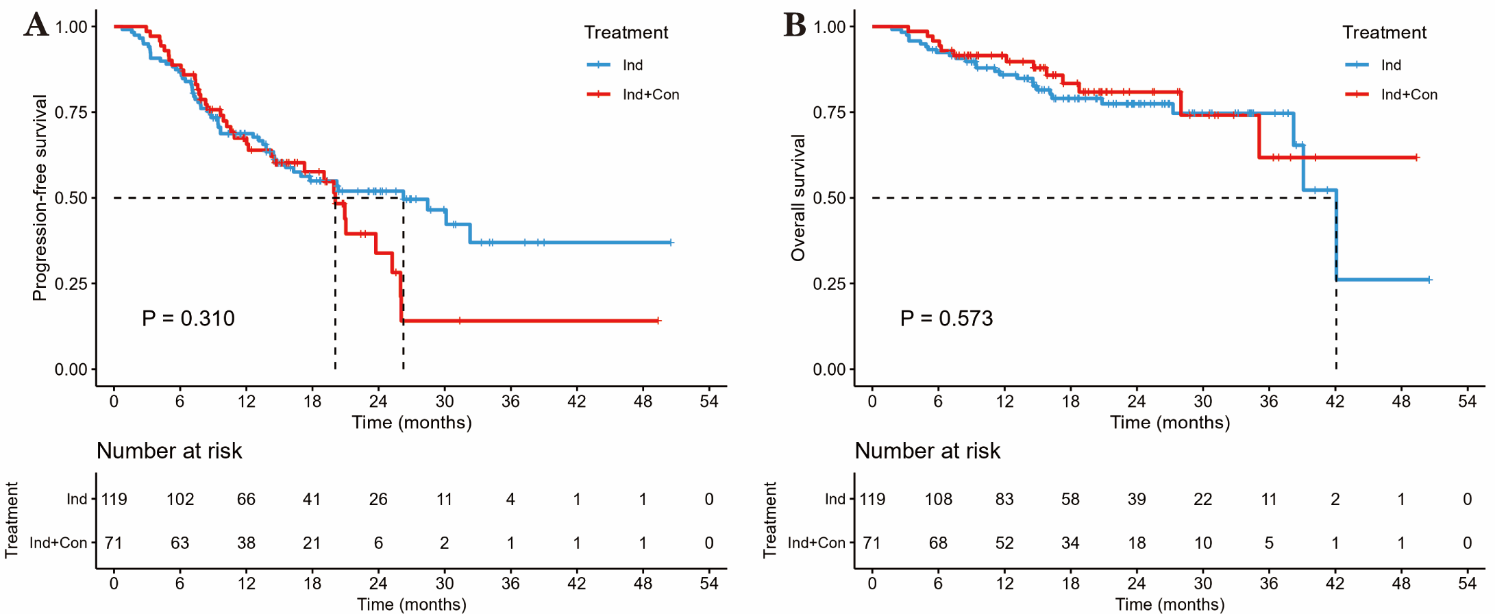


**Figure S4.** PFS and OS between the Ind and Ind+Con groups, measured from the initiation of radiotherapy. **(A)** PFS. **(B)** OS.


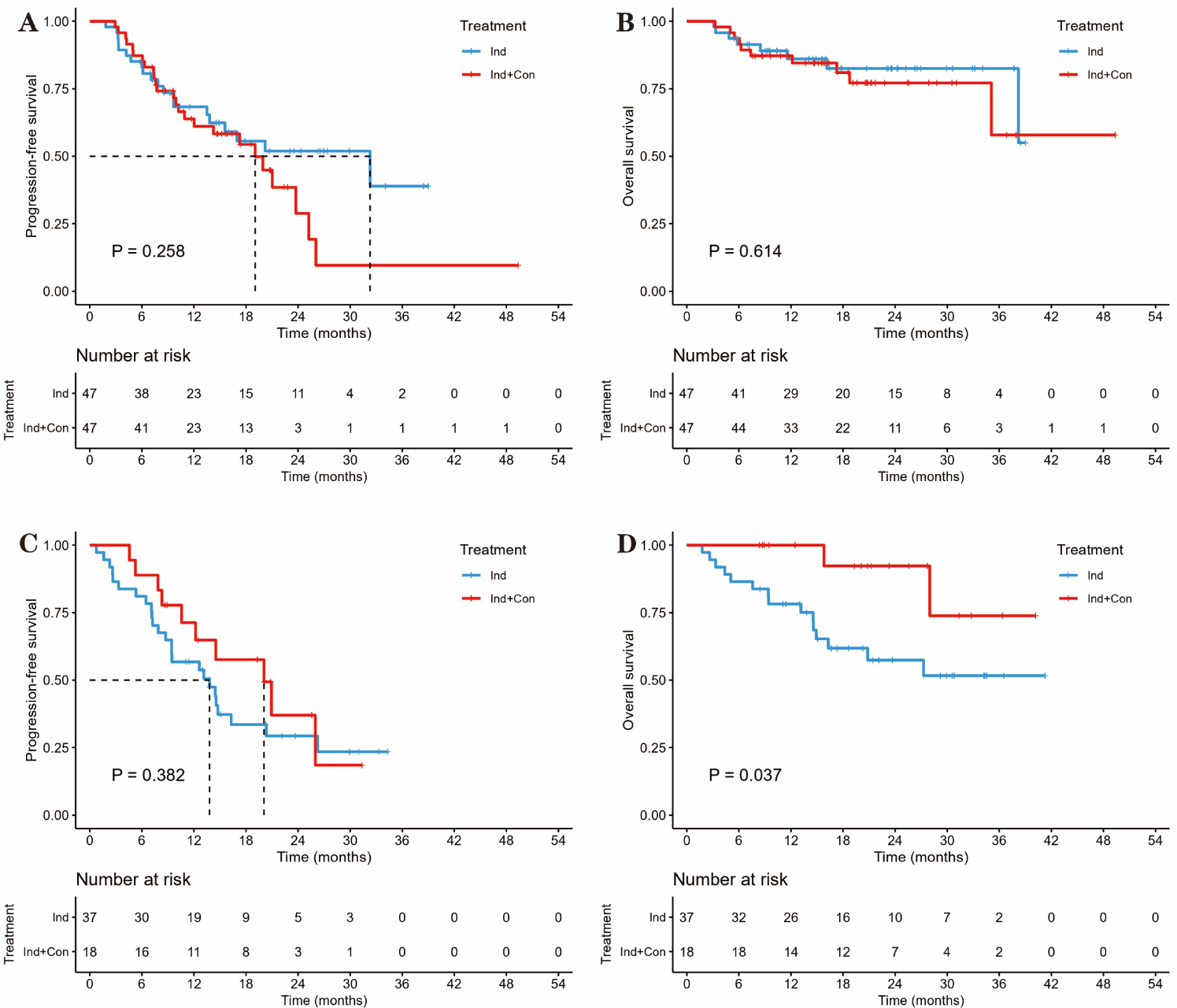


**Figure S5.** PFS and OS between the Ind and Ind+Con groups stratified by response to induction chemoimmunotherapy, measured from the initiation of radiotherapy. **(A)** PFS in patients with CR or PR. **(B)** OS in patients with CR or PR. **(C)** PFS in patients with SD or PD. **(D)** OS in patients with SD or PD.


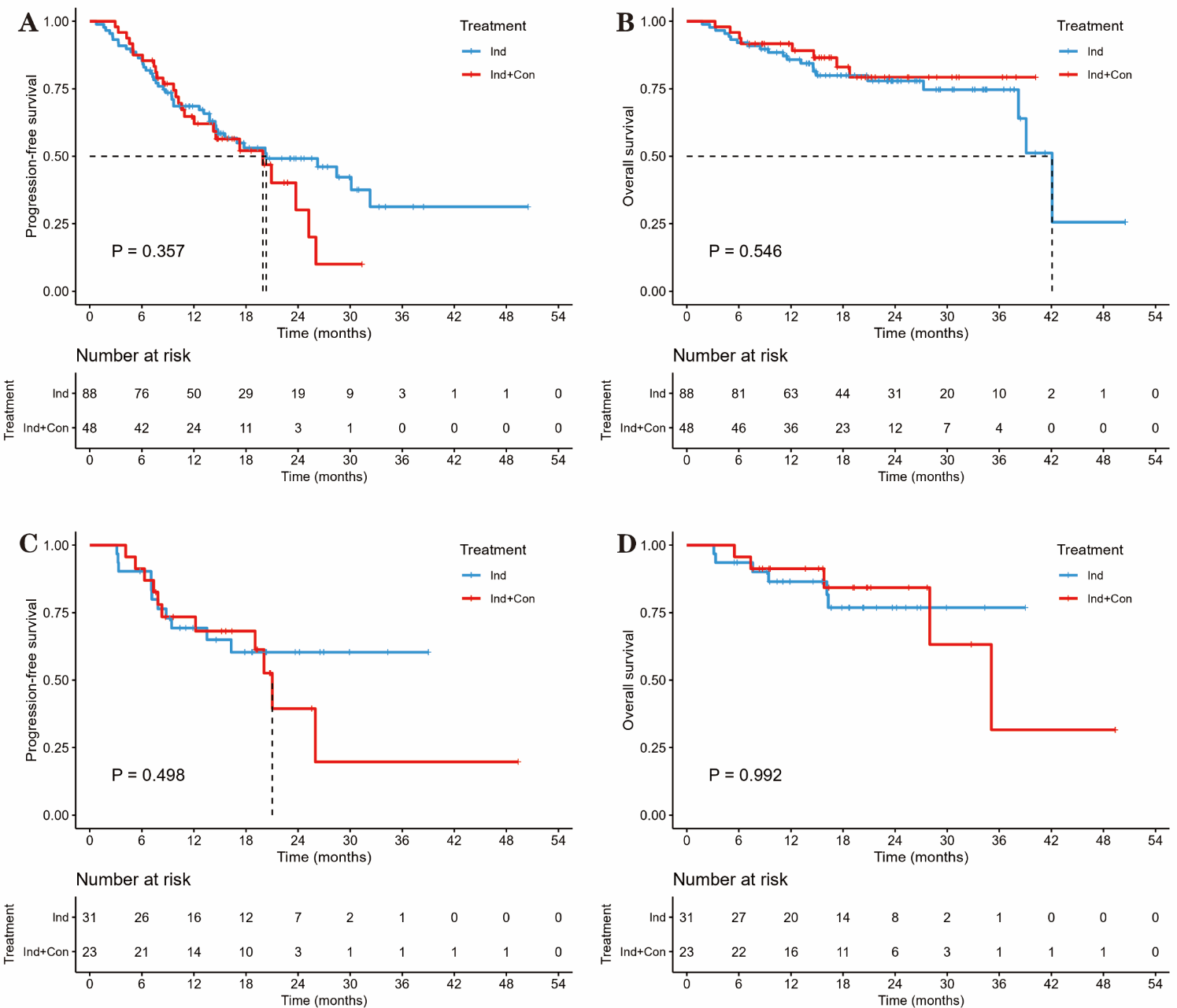


**Figure S6.** PFS and OS between the Ind and Ind+Con groups in the sequential or concurrent chemoradiotherapy subgroup, measured from the initiation of radiotherapy. **(A)** PFS in the sequential chemoradiotherapy subgroup. **(B)** OS in the sequential chemoradiotherapy subgroup. **(C)** PFS in the concurrent chemoradiotherapy subgroup. **(D) O**S in the concurrent chemoradiotherapy subgroup.
